# Great debate: the new risk factor–weighted clinical likelihood model is useful to estimate the initial pre-test probability of obstructive coronary artery disease in individuals with suspected chronic coronary syndromes

**DOI:** 10.1093/eurheartj/ehag091

**Published:** 2026-03-13

**Authors:** Felicita Andreotti, Holger Thiele, Christiaan Vrints, Alexander Jobs, Simon Winther, Jens Gröninger, Davide Capodanno, Steffen Desch, Sotirios Nedios, Diana A Gorog

**Affiliations:** CardioThoracic Department, Catholic University Medical School, Largo A. Gemelli 8, Rome 00168, Italy; Cardiovascular Science Department, Fondazione Policlinico Universitario Gemelli IRCCS, Largo Francesco Vito 1, 00168 Roma, Italy; Department of Internal Medicine/Cardiology, Heart Center Leipzig at Leipzig University, Strümpellstr. 39, Leipzig 04289, Germany; Research Group of Cardiovascular Diseases, GENCOR, University of Antwerp, Antwerp, Belgium; Department of Cardiology, Antwerp University Hospital, Edegem, Belgium; Department of Internal Medicine/Cardiology, Heart Center Leipzig at Leipzig University, Strümpellstr. 39, Leipzig 04289, Germany; Department of Cardiology, Gødstrup Hospital, Herning, Denmark; Institute of Clinical Medicine, Aarhus University, Aarhus, Denmark; Department of Internal Medicine/Cardiology, Heart Center Leipzig at Leipzig University, Strümpellstr. 39, Leipzig 04289, Germany; Division of Cardiology, Azienda Ospedaliero-Universitaria Policlinico G. Rodolico–San Marco, University of Catania, Catania, Italy; Department of Internal Medicine/Cardiology, Heart Center Leipzig at Leipzig University, Strümpellstr. 39, Leipzig 04289, Germany; Department of Internal Medicine/Cardiology, Heart Center Leipzig at Leipzig University, Strümpellstr. 39, Leipzig 04289, Germany; Faculty of Medicine, National Heart and Lung Institute, Imperial College, Dovehouse Street, London SW3 6LY, UK; Centre for Health Services and Clinical Research, Postgraduate Medical School, University of Hertfordshire, Hatfield, Hertfordshire AL10 9AB, UK; Cardiology Department, East and North Hertfordshire NHS Trust, Coreys Mill Lane, Stevenage, Hertfordshire SG1 4AB, UK; School of Cardiovascular and Metabolic Medicine & Sciences, Faculty of Life Sciences & Medicine, King's College London, 125 Coldharbour Lane, London, SE5 9NU, UK

**Keywords:** Coronary artery disease, Prediction, Risk factors, Clinical likelihood, Risk score, Symptoms, Prognosis

## Abstract

For individuals with suspected chronic coronary syndrome, the 2024 ESC guidelines recommend use of a structured estimate of the probability of obstructive coronary artery disease (CAD). This ‘risk factor–weighted clinical likelihood’ (RF-CL) model is recommended as the initial step after history taking and combines age, sex, symptom characteristics, and five clinical risk factors, with coronary calcification data, if available. The resulting numerical estimate indicates an initial ‘pre-test probability’ of obstructive CAD, that has been calibrated to provide improved accuracy compared with previous models. It can help triage patients for appropriate testing and identify individuals with a very low likelihood of obstructive CAD, for whom deferral of further diagnostic tests should be considered. Designed to assess the likelihood of obstructive CAD, the RF-CL model is not designed to predict ischaemia, which may occur in the absence of obstructive coronary disease and could account for patient symptoms. The score is easy and quick to use, with extensive external validation in contemporary populations including European, North American, and Asian cohorts. However, some have questioned the practical application of the RF-CL tool, citing challenges with the specificity and clarity of patient symptoms, definition and weighting of risk factors, as well as the other ‘enrichment factors’ that can enhance the likelihood. The RF-CL model is quantitative up to 45% and then becomes semi-quantitative/qualitative. For patients considered very high likelihood, with an estimated score > 85%, invasive coronary angiography is recommended, although how this score may be reached is not entirely clear. The RF-CL model undoubtedly improves the prediction of obstructive CAD and can ‘de-risk’ a significant number of symptomatic patients safely, reducing unnecessary testing. In the development and application of such a probability estimate, there is a need to strike a good balance between simplicity and usefulness, vs increased sensitivity at the expense of greater complexity. Here, the two sides of this Great Debate are presented, to help the reader better evaluate the practical usefulness of the new RF-CL assessment in predicting the probability of obstructive CAD.

## Graphical Abstract

**Graphical Abstract:**
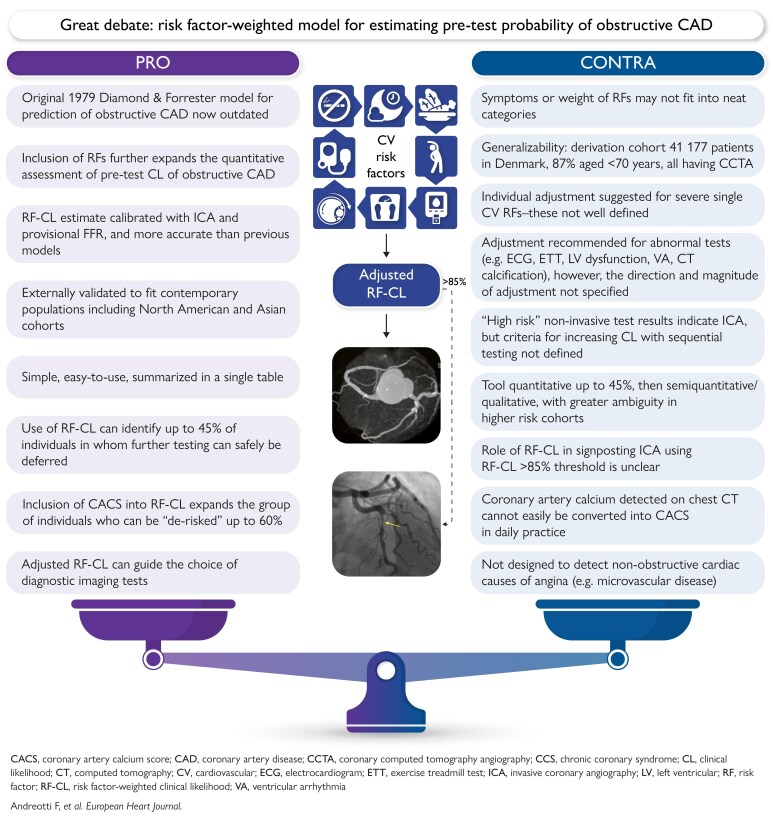
Key points in favour (left) or against (right) the new risk factor–weighted clinical likelihood (RF-CL) assessment employed in the 2024 ESC guideline in the initial assessment of individuals with suspected chronic coronary syndrome. Central panels show common cardiovascular risk factors, the adjusted RF-CL model, and tests employed to detect obstructive coronary artery disease, namely, coronary CT angiography and invasive coronary angiography.

*‘Medicine is a science of uncertainty and an art of probability’.* ^1^

Sir William Osler (1849–1919)

## Introduction

Estimating the ‘pre-test’ probability (PTP) of a condition can help triage patients for appropriate testing for that condition.

Learning and experience enable clinicians to make informed decisions about the likely cause of symptoms. Diagnosing a disease, even before any tests are undertaken (i.e. after history taking and examination), often involves considering multiple possibilities and weighing the likelihood of each. In essence, the practice of medicine involves a constant process of balancing probabilities, considering uncertainties, and making decisions based on the available relevant information and the most useful and precise course of action.

The diagnostic pathway of evaluating most clinical symptoms in medicine, including chest pain, centres on the probabilistic likelihood of a given disease, so that estimating the PTP of a condition can help triage patients for appropriate testing.

Firstly, an initial assessment, most often focused on symptoms, enables a broad differential diagnosis to be made. Next, based on individual patient characteristics, the clinician adjusts the probabilities of diseases, reflected in a patient-specific differential diagnosis. This is the initial PTP. Based on the PTP, one or more diagnostic tests are chosen to make a definite diagnosis. This principle has been used for decades by clinicians evaluating patients with chest pain in the outpatient setting.

Traditionally, the estimation of the PTP of obstructive coronary artery disease (CAD) was based on the 1979 Diamond and Forrester model, which relies on three basic characteristics of age, sex, and nature of symptoms to assess the likelihood of obstructive CAD. However, this simplified approach overestimates the PTP when used in contemporary patient populations, in whom CAD prevalence adjusted for age is lower than in historical cohorts, as lower probability patients are being referred.^2–4^

To account for this change in demographic, the 2019 European Society of Cardiology (ESC) guidelines for the diagnosis and management of chronic coronary syndromes (CCS)^5^recommended estimating the likelihood of CAD using a recalibrated Diamond–Forrester approach, taking into account coefficients of sex, age, and symptom-type, based on a pooled analysis of three large contemporary studies^6^ which used computed tomography (CT)^7,8^ or invasive coronary angiography^9^ as the reference standard. This enabled substantial ‘down-classification’, i.e. moving from a higher clinical likelihood (CL) to a lower adjusted CL category, compared with previous models.

Although the 2019 ESC guidelines floated the concept of CL to guide the selection of diagnostic tests, no specific tool was provided to estimate the CL.^5^

In 2024, the ESC guidelines for the management of CCS further refined the model for estimating the likelihood of CAD, based on the CL concept.^10^ In addition to age, sex, and the nature of the symptoms, this time the presence of risk factors was incorporated to estimate the PTP of obstructive CAD, termed the risk factor–weighted clinical likelihood model (RF-CL).

## Why do we need a new clinical likelihood assessment model?

The guiding principles for triaging individuals with tests include the need to (i) identify disease, including forms that require intervention, (ii) avoid unnecessary testing, and (iii) maximize cost-effectiveness.

It is mainly the last two aims that improved risk prediction models can address.

Firstly, the prevalence of significant CAD among patients referred for chest pain investigations has declined significantly in the last 30 years, such that prior PTP models overestimate the likelihood of CAD in contemporary cohorts, with consequences of overtesting and increased burden on healthcare systems and costs.^11,12^ Over-investigation can lead to unnecessary anxiety, or potentially increased risks associated with testing.

Secondly, expansion in availability and refinement of different diagnostic test modalities in the last few decades, including coronary artery calcium score, coronary CT angiography, CT fractional flow reserve, stress echo, cardiovascular magnetic resonance, and positron emission tomography, has focused attention on directing patients to the most appropriate test(s).

Thirdly, there is a pressing need to maximize the yield of diagnostic testing in the setting of limited resources.

## Why should we use the risk factor–weighted clinical likelihood model tool when assessing patients with chest pain?

The RF-CL tool adopted by the 2024 ESC CCS guideline is easy and quick to use, requiring only data available from simple history taking.

Compared with probability assessment without clinical risk factors, the RF-CL has been shown to improve the prediction of obstructive CAD, down-classifying a significant number of individuals to a very low likelihood category who can therefore avoid further testing, and has been validated in large cohorts.^12,13^

A diagnostic test should generally only be performed when its result could alter management. Recent studies have shown that the RF-CL assessment has superior predictive and discriminatory performance, compared with previous models. Finally, incorporation of the tool into clinical practice would be expected to lead to substantial cost savings compared with earlier PTP models because 27% more patients with the RF-CL model would be deferred from testing. These and other model advantages are discussed in the Pro section.

## What are the potential concerns about using the risk factor–weighted clinical likelihood model tool when assessing patients with chest pain?

There are general and specific concerns about using the RF-CL tool. Generally, in every day clinical practice, patients may not fit neatly into clearly defined categories, either in the nature of their reported symptoms or in the weight of risk factors (e.g. from the patient with recent diagnosis of mildly impaired glucose tolerance to the patient with long-standing poorly controlled diabetes). Additionally, there is always a concern that recommended use of the RF-CL model is misunderstood as a rule rather than a guide.

However, there are also specific potential concerns about the RF-CL tool.

The RF-CL model estimates the probability of obstructive CAD (expressed as %) based on symptoms, risk factors, age, and sex (Figure 4 of 2024 ESC CCS guideline). The risk scoring is quantitative up to 45%, and then, it becomes semi-quantitative/qualitative, with greater ambiguity in higher likelihood cohorts. These aspects are discussed in the Contra section.

The 2024 ESC CCS guideline recommends that the RF-CL estimate be adjusted in the presence of any abnormal clinical findings, namely, resting ECG changes (Q-wave or ST-segment/T-wave changes), exercise ECG with abnormal findings, left ventricular dysfunction (severe or segmental), ventricular arrhythmia, peripheral artery disease, or coronary calcification on pre-existing chest CT.

However, use of these ‘enrichment factors’ which increase the likelihood of CAD is not immediately straightforward, given a lack of detail about what constitutes each abnormal clinical finding. For example, is non-specific lateral strain pattern on the 12-lead ECG in a patient with long-standing hypertension sufficient to add to the RF-CL model risk assessment?

The guideline also states that ‘individual adjustment of the likelihood may be necessary for individuals with severe single risk factors or comorbidities associated with an increased prevalence of obstructive CAD, which are not reflected in the RF-CL model’. While some examples are given, such as familial hypercholesterolaemia, severe kidney dysfunction, or rheumatic/inflammatory diseases, these remain poorly defined. The ‘enrichment’ factors are not weighted, and it is unclear whether their risk is additive in gauging overall risk.

However, while avoiding unnecessary tests is clearly desirable, decisions regarding prevention may be affected by diagnostic testing, which may be denied if disease is not detected.

In the patient with chest pain, the assessment of the likelihood of obstructive CAD is only one part of the CCS diagnostic algorithm. The RF-CL tool is not designed to predict non-obstructive disease, but rather to estimate the likelihood of obstructive CAD, a clinical condition whose prognosis potentially can be improved by revascularization. With increased awareness that angina can occur in the absence of obstructive CAD, e.g. due to microvascular dysfunction and vasospasm, should we focus on the detection of ischaemia, rather than obstructive epicardial disease?

The RF-CL model undoubtedly improves the prediction of obstructive CAD, compared with earlier models and can ‘de-risk’ a significant number of symptomatic patients safely.^14^ However, it is important to involve patients in decisions regarding their care, including whether or not further testing is performed, with informed choice about the pros and cons of different strategies, to enable shared decision-making. There are inherent limitations to using any score and striking a balance between simplicity and usefulness, vs increased sensitivity at the expense of greater complexity.

It should be borne in mind that while RF-CL was shown to improve the prediction of obstructive coronary disease, with an improved C-statistic compared with the simple PTP model, the discriminatory ability of the score had an area under the receiver operating curve of 75 (95% confidence interval 74–76).^13^ Ultimately, it is a guide. It is probably the best we have.

**Figure 1:**
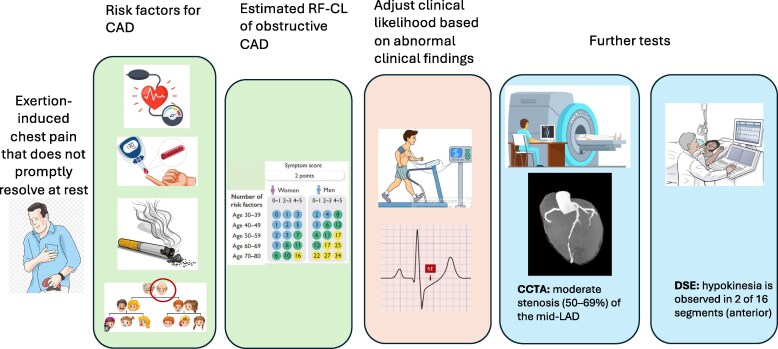
Case example. A 53-year-old man presents with exertion-induced constricting chest pain that does not typically resolve promptly at rest (European Society of Cardiology symptom score = 2). He has four cardiovascular risk factors: a family history of coronary artery disease (his father had a myocardial infarction at 49 years of age), smoking, hypertension, and diabetes mellitus. Based on the 2024 European Society of Cardiology guidelines for the management of chronic coronary syndromes, the risk factor–weighted clinical likelihood is 17%. An exercise ECG shows ST-segment depression at a moderate workload, which may qualify as an abnormal clinical finding according to the guidelines (see Figure 5 of the guideline document) and could prompt adjustment of the risk factor–weighted clinical likelihood model. To further evaluate the likelihood of obstructive coronary artery disease, additional non-invasive tests are performed. A coronary computed tomography angiography reveals single vessel disease with moderate stenosis (50%–69%) of the mid-left anterior descending artery. Subsequently, a dobutamine stress echo shows hypokinesia in 2 of 16 segments (anterior). The ‘debaters’ will discuss the approach to using the risk factor–weighted clinical likelihood model as applied to this case example

**Figure 4:**
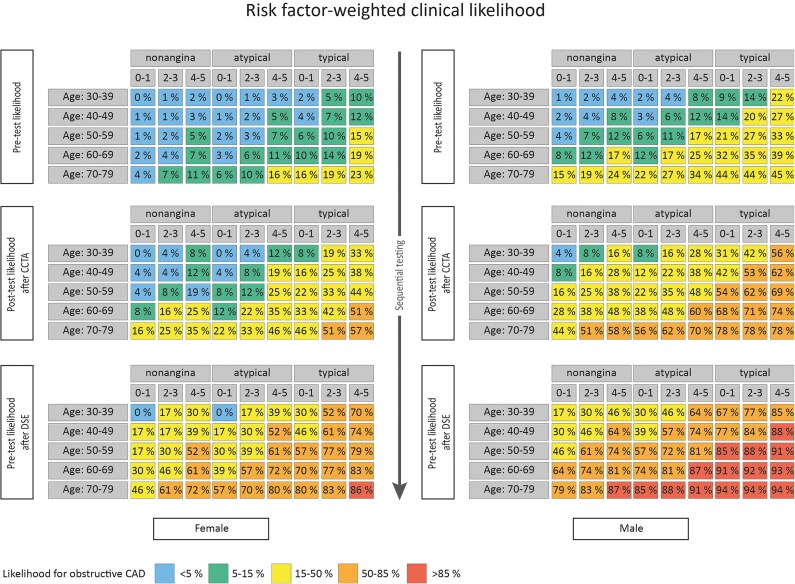
Tabulated (rigid mathematical) likelihood of obstructive coronary artery disease according to the risk factor–weighted clinical likelihood as published by Winther *et al*.^4^ (upper section), after a positive coronary computed tomography angiography (middle section), and after an additional positive dobutamine stress echocardiography (lower section). It is evident that a clinical likelihood >85%, which qualifies for invasive coronary angiography, is only reached in very few cases

Here we present the two sides of the debate, from the Pro and the Contra sides (*Graphical Abstract*). We present a case example (*Figure 1*) and the ‘debaters’ will discuss the approach to using the RF-CL as applied to this case.

We hope this debate will enable the reader to better evaluate the usefulness of the new RF-CL assessment in predicting the likelihood of obstructive CAD.

## Supplementary Material

ehag091_Supplementary_Data

## Figures and Tables

**Figure 2:**
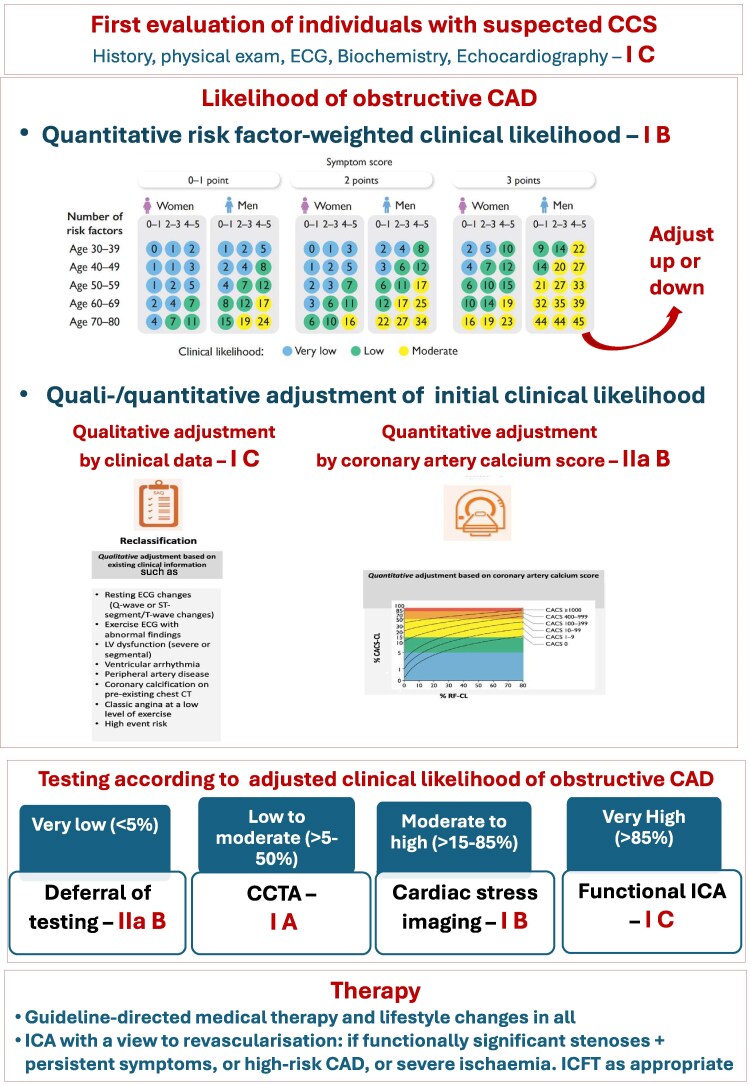
The 2024 European Society of Cardiology model to estimate the initial ‘pre-test’ clinical likelihood of obstructive coronary artery disease in individuals with suspected chronic coronary syndrome. After initial evaluation, calculate the risk factor–weighted clinical likelihood and adjust it using available clinical and coronary calcium information. Decide on further testing according to the adjusted clinical likelihood. Treat according to guidelines. Assessing the likelihood of obstructive coronary artery disease is only part of the 2024 guidelines’ diagnostic algorithm. Non-obstructive forms of chronic coronary syndrome should also be considered. The initial ‘pre-test’ clinical likelihood of obstructive coronary artery disease is not the only indication for invasive coronary angiography. CACS, coronary artery calcium score; CAD, coronary artery disease; CCS, chronic coronary syndrome; CCTA, coronary computed tomography angiography; CL, clinical likelihood; ECG, electrocardiogram; ICA, invasive coronary angiography; ICFT, invasive coronary functional testing; LV, left ventricular; RF-CL, risk factor–weighed clinical likelihood. I and IIa refer to class of recommendation and A, B and C to level of evidence. Details in text and guidelines^1^

**Figure 3:**
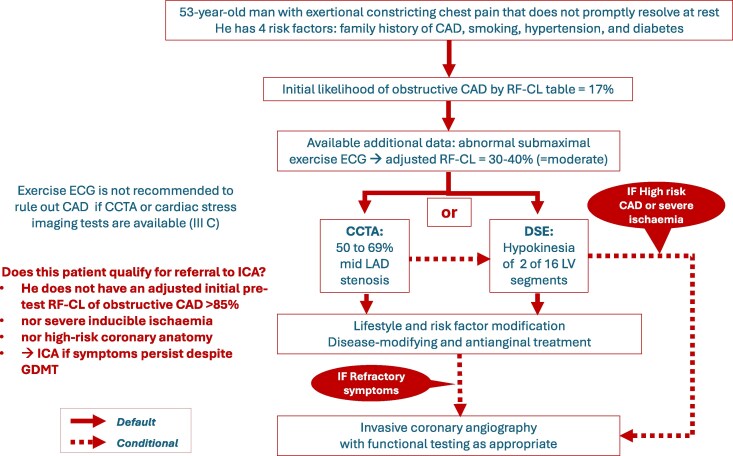
Guideline-directed diagnostic algorithm applied to the clinical vignette. CAD, coronary artery disease; CCTA, coronary computed tomography angiography; DSE, dobutamine stress echocardiography; ECG, electrocardiogram; GDMT, guideline-directed medical therapy; ICA, invasive coronary angiography; ICFT, invasive coronary functional testing; LAD, left anterior descending artery; LV, left ventricular; RF-CL, risk factor–weighted clinical likelihood. III refers to class of recommendation and C to level of evidence

## Pro

https://orcid.org/0000-0002-1456-6430AndreottiFelicita
CardioThoracic Department, Catholic University Medical School, Largo A. Gemelli 8, Rome 00168, Italy
Cardiovascular Science Department, Fondazione Policlinico Universitario Gemelli IRCCS, Largo Francesco Vito 1, 00168 Roma, Italyhttps://orcid.org/0000-0002-9263-5702VrintsChristiaan
Research Group of Cardiovascular Diseases, GENCOR, University of Antwerp, Antwerp, Belgium
Department of Cardiology, Antwerp University Hospital, Edegem, Belgiumhttps://orcid.org/0000-0001-8872-3681WintherSimon
Department of Cardiology, Gødstrup Hospital, Herning, Denmark
Institute of Clinical Medicine, Aarhus University, Aarhus, Denmark

* Corresponding author. Email: felicita.andreotti@unicatt.it

† Felicita Andreotti, Christiaan Vrints and Simon Winther contributed equally.

### The 2024 European Society of Cardiology guideline-directed estimate of obstructive coronary artery disease (*Figure 2*)

For individuals presenting with suspected chronic coronary syndrome (CCS), the 2024 ESC guidelines recommend a structured estimate of the likelihood of obstructive epicardial coronary artery disease (CAD) (class I, evidence B).^1^Obstructive CAD is defined as a diameter stenosis ≥50% at coronary angiography.

The process involves reading a half-page table that considers—in addition to symptoms, sex, and age—five risk factors, yielding a numerical initial likelihood of obstructive CAD, with a maximum value of 45%.^1^ The use of coronary calcium data, if known, is encouraged to refine the numerical likelihood (class IIa, evidence B).^1^ Given lack of contemporary mathematical models, clinicians must qualitatively/semi-quantitatively integrate any other relevant information to further refine the estimate (class I, evidence C).^1^ Through adjustments, the likelihood may exceed the table’s maximum.

Assessing the likelihood of obstructive CAD is only part of the guidelines’ diagnostic algorithm. Non-obstructive forms of CCS should also be considered.^1^

### European Society of Cardiology guidelines

European Society of Cardiology guidelines are overseen by a dedicated committee, 100 external reviewers, and ESC staff. Each recommendation is supported by available evidence (for levels A and B) and undergoes three voting rounds by a Task Force, which must comply to strict standards. The 2024 CCS guidelines include 351 pages of evidence tables and 196—mostly strong—recommendations (162 class I or IIa and 142 evidence A or B).^1^

### Previous scores

With population aging and improved survival, cardiovascular disease remains a global burden and ischaemic heart disease the leading cause of death.^2–5^ In parallel, the diagnostic yield of anatomical and functional tests for obstructive CAD has declined,^6–10^ reflecting a lower age-adjusted population prevalence^11–14^ and a shift from high-risk patients, previously sent directly to invasive coronary angiography (ICA), towards a broader, lower-risk population^15^ now evaluated non-invasively with coronary computed tomography angiography (CCTA). Thus, the 1979 Diamond and Forrester model^11^—though long used—greatly overestimates contemporary CAD probability. Successive refinements—including the updated Diamond–Forrester^16^ and CAD Consortium scores,^17^ and the 2019 ESC model^18,19^—provide lower, more accurate estimates for a given combination of variables.^11,16–18^ The 2019 ESC model calibrates well against CCTA/ICA, but still slightly overestimates observed disease.^20^

Notably, the 2019 ESC CCS guidelines introduced the ‘clinical likelihood’ concept, marking an evolution from a simple quantitative probability table to a more qualitative, comprehensive initial estimate of obstructive CAD likelihood.^19^ This approach integrates not only age, sex, and symptoms, but also risk factors, comorbidities, coronary calcium, cardiac electrical, mechanical or perfusion abnormalities, and any other useful available data, capturing the true clinical context, rather than relying on rigid, non-exhaustive ‘pre-test’ probabilities.^19^

### The risk factor–weighted assessment

The risk factor–weighted clinical likelihood (RF-CL) table recommended by the 2024 ESC guidelines is an advancement over previous models because it integrates age, sex, symptom characteristics (quality/location, triggers, and resolution), and risk factors (family history, past or current smoking, dyslipidaemia, hypertension, and diabetes) with coronary calcium data, if available, into a single score that is a numerical initial ‘pre-test’ estimate of obstructive CAD. The score has been calibrated and validated against angiography in contemporary Western and Asian populations,^1,21–29^ with incremental accuracy compared with the 2019 model.^20,25^

Estimating the RF-CL of obstructive CAD is the initial step after history taking and basic tests. It helps select or defer downstream diagnostic tests (*Figure 2*).^1^ In most Western cohorts with suspected CCS, the RF-CL yields very low (≤5%), low (>5%–15%), or moderate (>15%–50%) likelihoods of obstructive CAD.^26,29^ Importantly, compared with previous models, the RF-CL model has an increased ability to reliably identify individuals with a very low likelihood,^26^ for whom the 2024 ESC guidelines state that ‘deferral of further diagnostic tests should be considered’ (class IIa, evidence B), limiting follow-up to the treatment of symptoms, comorbidities, lifestyle, and risk factors.^1^

### Disease prediction, prognostic value, and guide to further testing

The RF-CL table was developed in a cohort of 41 177 symptomatic individuals undergoing first-time CCTA.^26^ The initial machine learning model, including body mass, renal function, and comorbidity index, underwent stepwise simplification without losing accuracy. Incorporating risk factor data further improved performance.

External validation in 15 411 Danish and North American patients demonstrated superior discrimination in predicting obstructive CAD compared with the 2019 ESC model [area under the curve (AUC) 0.78, 95% confidence interval (CI) 0.77–0.79 vs 0.76, 95% CI 0.75–0.76; *P* < .05].^26^ Other strengths include improved disease prediction after integration of coronary calcium data,^21^ close agreement with fractional flow reserve (FFR) at ICA,^27^ increased downward reclassification (38% very low likelihoods vs 11% with the 2019 model),^26^ and superior calibration and discrimination for FFR-defined obstructive CAD compared with the 2019 model.^27^ Further validation in different cohorts, including Asians, has led to AUCs of 0.73–0.87, highest values reached with calcium score inclusion.^22,24,28,29^ The RF-CL tends to underestimate >50% stenoses at CCTA^22,24,28,29^ but overestimate ischaemia on cardiac stress imaging.^30^

Longitudinal analyses in 45 000 patients showed clear gradients in the 5-year rates of adverse events among very low, low, moderate, and >50% RF-CL patient categories: 0.8%, 1.7%, 3.0%, or 7.0% for non-fatal myocardial infarction (MI) and 1.5%, 3.5%, 7.1%, or 14.5% for all-cause death, respectively.^21^ These results were generally confirmed in independent cohorts, including 5000 Chinese patients with stable chest pain.^22,24,29^

Finally, the RF-CL model is clinically useful in selecting downstream testing. In >13 000 PROMISE and SCOT-HEART trial participants, usual care was compared with initial CCTA. Although the overall composite of MI and death was only numerically lower in the CCTA arm,^31^ participants with low RF-CL (>5%–15%) had significantly lower event rates when tested with CCTA (HR 0.67, 95% CI 0.47–0.97).^31^

### Qualitative adjustment of the initial disease estimates

Any relevant information—including previously available test results—should be used to adjust the initial RF-CL of obstructive CAD, using knowledge and experience, to reach an ‘adjusted’ clinical likelihood (class I, evidence C).^1^ According to Bayes’ conditional analysis, no clinical information is independent of another, such that the likelihood after each investigation becomes the ‘pre-test’ likelihood of the next.^11^ Conditional analysis is used in everyday practice and involves a reiterative qualitative/semi-quantitative probabilistic approach, still largely relying on clinical expertise.^1,19^ Given infinite possible variables and permutations characterizing each patient, the 2024 guidelines intentionally provide a broad recommendation to ‘adjust clinical likelihood based on clinical findings’.^1^ The process may lead to lowering or increasing the value produced by the RF-CL table (*Figure 2*).

### Disease prediction and prognostic value of coronary artery calcium

Coronary artery calcium (defined on non-cardiovascular chest CT as none, mild, moderate or severe,^32^ or quantified more precisely from non-contrast, ECG-gated, coronary CT scans) has exceptionally high negative predictive values for obstructive CAD (>95%).^33^ Integrating calcium information into the RF-CL model improves discrimination vs calcium alone or other predictive models.^23,26^ After calcium integration, 54% of stable chest pain individuals can be classified as having a very low likelihood of obstructive CAD vs 38% using the RF-CL alone.^26^ Very high calcium scores (≥400 Agatston units) can reclassify initial RF-CLs to high (>50%–85%) or very high (>85%) likelihoods, i.e. well beyond the maximum 45% value of the RF-CL table (*Figure 2*).^26^ Calcium integration additionally improves risk stratification for incident MI and death compared with other models.^21^ Among stable chest pain individuals, the absence of coronary calcium allowed safe deferral of downstream testing, faster diagnoses, lower diagnostic costs, and no increase in event rates during follow-up (albeit with higher radiation) compared with no calcium evaluation.^22,24,33^

### Downstream and sequential testing

For patients with a low to moderate adjusted ‘pre-test’ RF-CL of obstructive CAD, the SCOT-HEART trial supports CCTA as the preferred diagnostic test, given its high ability to rule out disease and, importantly, its associated long-term reduction in coronary death and MI, compared with usual care involving exercise ECG.^34,35^ Coronary computed tomography angiography also provides unique non-invasive information on vessel wall and plaque characteristics. For those with moderate to high clinical likelihood, cardiac stress imaging is preferred, as supported by the PROMISE trial,^10^ owing to its high ability to rule in functionally significant obstructive CAD.^36^

Invasive coronary angiography is recommended in individuals with a very high (>85%) initial ‘pre-test’ RF-CL of obstructive CAD. This indication refers to *clinical* likelihood, adjusted individually based on all available clinical data *before* non-invasive imaging, not to post-imaging (or ‘post-test’) likelihood. Additional guideline-directed indications for ICA are (i) persistent symptoms despite guideline-directed medical therapy (GDMT); (ii) anginal symptoms at low exertional levels; (iii) other conditions associated with high risk of adverse events; and (iv) endotyping of angina/ischaemia with non-obstructed coronary arteries.^1^ The indication for ICA when symptoms persist despite GDMT should include haemodynamic assessment of stenosis severity and—in the absence of significant stenoses—testing to evaluate the contribution of vascular spasm and/or dysfunction to the mechanism of inducible ischaemia.

The 2024 ESC guidelines—in line with previous ones^19^—consistently and exclusively use ‘clinical likelihood’ when referring to the initial ‘pre-test’ estimate of obstructive CAD, as opposed to ‘post-test likelihood’ used to indicate the well-known relation between obstructive CAD prevalence and diagnostic test likelihood.^1^

Routine use of non-invasive sequential testing is not guideline-recommended.^1^ Only with inconclusive results of the first non-invasive imaging test, current guidelines—consistent with previous recommendations^19^—recommend further non-invasive imaging.^1^ Given CCTA’s limited rule-in capacity, additional testing is important when intermediate stenoses are detected at CCTA, to confidently rule in functionally significant obstructive CAD.^36^

The need for sequential imaging is generally limited. In a Finnish population, CCTA alone ruled out obstructive CAD in roughly half of patients with suspected CCS, thereby conferring a favourable prognosis.^37^ In the SCOT-HEART trial, about a quarter were found to have obstructive disease with stenoses exceeding 70%.^38^ Registry studies confirm that only a small proportion of individuals with suspected CCS proceeds to cardiac stress imaging (7%) or ICA (17%).^39,40^

Multiple studies^41–43^ show superior diagnostic accuracy of CCTA-derived FFR (FFR-CT) over CCTA alone, compared against invasive FFR. On meta-analysis, a negative FFR-CT predicted low 12-month event rates with significantly lower mortality and MI rates compared with positive results.^44^ Two randomized trials demonstrated that use of selective FFR-CT in ∼40% of patients reduced unnecessary ICAs without increasing events.^45,46^ Nationwide implementation data confirmed reduced downstream testing.^47^ Overall, pending availability, FFR-CT appears a viable alternative to traditional sequential imaging.^1^

### Applying the 2024 European Society of Cardiology guidelines to the contra clinical vignette (*Figure 3*)

A 53-year-old man presenting with exertional, constricting chest pain that does not promptly resolve at rest is suspected of angina pectoris. The patient has four major risk factors for premature obstructive CAD, supporting immediate lifestyle changes and strict risk factor control.

#### Determine the initial risk factor–weighted clinical likelihood model for obstructive coronary artery disease

Based on age, sex, number of risk factors, and symptom characteristics, the initial RF-CL is 17%, indicating a moderate likelihood of obstructive CAD.

#### Adjust the initial risk factor–weighted clinical likelihood model based on relevant available information

An available exercise ECG shows ST-segment depression at moderate workload, suggesting inducible myocardial ischaemia for less than maximal exertion. Myocardial ischaemia in this patient could result from obstructive CAD and/or microvascular dysfunction secondary to hypertension, smoking, and diabetes. Although an abnormal exercise ECG is associated with adverse outcomes,^1,48^ its diagnostic value to rule in or rule out obstructive CAD is limited compared with other tests.^36^ In this case, the abnormal, submaximal exercise ECG reasonably prompts a semi-quantitative increase of the 17% initial RF-CL to an adjusted RF-CL of 30%–40%.

#### Diagnostic testing with non-invasive imaging

Given the moderate adjusted clinical likelihood of obstructive CAD, either CCTA or cardiac stress imaging may be chosen as the next test (*Figures 2* and *3*). In this patient, both tests were prescribed. Coronary computed tomography angiography revealed an intermediate mid-left anterior descending (LAD) artery stenosis, and cardiac stress imaging demonstrated inducible hypokinesia of two anterior segments. Each test alone supports the diagnosis of angina pectoris, prompting antianginal therapy and single antiplatelet therapy, in addition to lifestyle changes and risk factor control.

#### When to consider invasive coronary angiography

A proximal LAD diameter stenosis ≥70% (with FFR ≤0.80) would indicate a high event risk (guideline recommendation *Table 14*, class I, evidence A).^1^ Since the mid-LAD stenosis in this patient was estimated at 50%–69%, additional testing may clarify its haemodynamic importance.

Dobutamine stress echocardiography showed inducible hypokinesia in two anterior segments, indicating inducible—but not high-risk—ischaemia. Although coronary microvascular dysfunction cannot be excluded as a *contributing* factor to ischaemia, hypokinesia in three segments during stress echocardiography would, by itself, be a marker of high event risk (guideline recommendation *Table 14*, class I, evidence B),^1^ justifying referral for ICA while proceeding to all GDMT.

This patient did not meet the high-risk CAD or ischaemia criteria that support early referral for ICA and revascularization. Therefore, through shared decision-making with an informed patient, the effects of GDMT can be monitored over time, and if symptoms persist despite optimized therapy, ICA may then be considered.

### Summary and conclusions

The RF-CL model is based on a recalibrated Diamond-Forrester score supplemented by risk factors and, optionally, coronary calcium scoring. It takes quantification of a ‘pre-test’ probability of obstructive CAD further downstream. The general principle that the assessment has a score-based, quantitative element and an additional arbitrary element, based on clinical judgement, is the same in both 2019 and 2024 ESC guidelines.^1,19^

The RF-CL model is simple and validated. Summarized in a single table, it outperforms previous models in predicting presence of obstructive CAD and long-term adverse events; sharing the resulting score with the patient enhances patient engagement, shared decision-making, and choice of downstream tests. The RF-CL model can reduce the number of initial tests by identifying more patients with very low (≤5%) obstructive CAD likelihood.^21^ Two randomized trials are further investigating deferred testing based on RF-CL (PERMI NCT06708000 and OPERATE NCT05640752).^49^ For adjusted likelihoods >5%, a single downstream test often suffices, but sequential testing should be considered if results are inconclusive. The recommendation for ICA based on the adjusted RF-CL does not apply to ‘post-imaging test probabilities’, where severity of ischaemia, high-risk coronary anatomy, and refractory symptoms are the major drivers.

Well-validated scores provide easy tools to predict, in a systematic, structured, and fairly reproducible way, disease likelihoods and adverse event risk categories. With patient education and shared decision-making at the forefront, physicians are encouraged to think probabilistically, drawing upon their expertise in interpreting patient data and treatment response.

## Contra

JobsAlexander
Department of Internal Medicine/Cardiology, Heart Center Leipzig at Leipzig University, Strümpellstr. 39, Leipzig 04289, GermanyGröningerJens
Department of Internal Medicine/Cardiology, Heart Center Leipzig at Leipzig University, Strümpellstr. 39, Leipzig 04289, Germanyhttps://orcid.org/0000-0002-5156-7723CapodannoDavide
Division of Cardiology, Azienda Ospedaliero-Universitaria Policlinico G. Rodolico—San Marco, University of Catania, Catania, Italyhttps://orcid.org/0000-0003-4222-6633NediosSotirios
Department of Internal Medicine/Cardiology, Heart Center Leipzig at Leipzig University, Strümpellstr. 39, Leipzig 04289, Germanyhttps://orcid.org/0000-0002-1349-9969DeschSteffen
Department of Internal Medicine/Cardiology, Heart Center Leipzig at Leipzig University, Strümpellstr. 39, Leipzig 04289, Germanyhttps://orcid.org/0000-0002-0169-998XThieleHolger
Department of Internal Medicine/Cardiology, Heart Center Leipzig at Leipzig University, Strümpellstr. 39, Leipzig 04289, Germany

*Corresponding author. Tel: +49 341 865 1428, Fax: +49 341 865 1461, Email: alexander.jobs@medizin.uni-leipzig.de

### Introduction

On 30 August 30 2024, the European Society of Cardiology (ESC) published updated guidelines for the management of chronic coronary syndromes (CCS).^1^ In these guidelines, a stepwise approach for the evaluation, confirmation of diagnosis, and risk estimation of individuals with suspected CCS has been introduced. Estimating the clinical likelihood (CL) of obstructive coronary artery disease (CAD) is central to this process, as it guides the selection of diagnostic tests. In the first step, the risk factor–weighted clinical likelihood (RF-CL) is estimated based on the quality of symptoms, the patient's sex and age, as well as the number of risk factors for obstructive CAD as described in the Introduction of this *Great Debate*. The maximum likelihood achievable with the RF-CL chart is 45%. In a second step, the ESC guidelines recommend adjusting the RF-CL based on abnormal clinical findings. Depending on the resulting adjusted RF-CL, further diagnostic assessment may be undertaken to confirm or exclude obstructive CAD. For an (adjusted) RF-CL between >5% and ≤85%, non-invasive tests such as coronary computed tomography angiography (CCTA), cardiac magnetic resonance (CMR) imaging, dobutamine stress echocardiography (DSE), or single-photon emission computed tomography (SPECT) should be performed. First-line invasive coronary angiography (ICA) is recommended in patients with high-risk CAD on CCTA, severe ischaemia on functional imaging, refractory angina, or a CL above 85%.

Generally, we support the new RF-CL model for estimating the initial pre-test likelihood of obstructive CAD and the subsequent stepwise diagnostic approach proposed by the ESC guidelines. However, as outlined in more detail below, its presentation as a quantitative, evidence-based approach may warrant clarification, as it incorporates both quantitative and qualitative components that can be difficult to implement consistently in clinical practice.

### Step 1: Estimate the risk factor–weighted clinical likelihood of obstructive coronary artery disease

In 1979, Diamond and Forrester laid the foundation for estimating the CL of obstructive CAD, using age-, sex-, and symptom-based data. Their conditional probability analysis became a reference point, though they already cautioned that their tables were only preliminary. More than 30 years later, the approach was revisited, and the CL model was updated using data from contemporary cohorts, by the CAD Consortium (2011), Juarez-Orozco *et al*. (2019), and Winther *et al*. (2020), whose models have since been adopted into subsequent ESC guidelines.^2–4^ It is immediately noticeable that the CL estimates are lower in the more recent cohorts (*Figure 4*; Supplementary data online, *Figure S1*). However, it is also important to emphasize that this does not reflect the prevalence of CAD in the general population, which has actually increased, likely due to improved survival rates resulting from advances in the treatment and secondary prevention of CAD, as well as the aging populations in many countries.

However, the CL of patients with chest discomfort may have decreased, likely as a result of healthier lifestyles (e.g. reduced smoking) and better management of cardiovascular risk factors in primary prevention (e.g. hypertension). Furthermore, a lower threshold to seek medical consultation for chest discomfort may have contributed to the observed decrease in CL, representing a form of ascertainment bias.

The RF-CL model used in the current guidelines is derived from a cohort of 41 177 patients who underwent first-time CCTA between 2008 and 2017 at 13 hospitals participating in the Western Denmark Heart Registry. Importantly, patients who primarily underwent ICA were not included.^4^ Of note, only 13% of patients in the derivation cohort were older than 70 years.^4^ Consequently, the CL estimates for this age group may be imprecise. Although it may be open to discussion whether the probability of obstructive CAD from a single Northern European chest pain population is generalizable to other geographical areas, the RF-CL model has undergone external validation in the Dan-NICAD, PROMISE, and SCOT-HEART populations.^4–6^

### Step 2: adjust clinical likelihood based on abnormal clinical findings

Based on the estimated pre-test probability, the 2024 ESC guidelines recommend in *Figure 5* adjusting the RF-CL estimate according to abnormal clinical findings, including

- Resting ECG changes (Q-wave or ST-segment/T-wave changes)
- Exercise ECG with abnormal findings
- Left ventricular (LV) dysfunction (severe or segmental)
- Ventricular arrhythmia (VA)
- Peripheral artery disease
- Coronary calcification on pre-existing chest CT

However, the specific recommendation in *Table 3* of the guidelines states: ‘It is recommended to use additional clinical data (e.g. examination of peripheral arteries, resting ECG, resting echocardiography, presence of vascular calcifications on previously performed imaging tests) to adjust the estimate yielded by the Risk Factor–weighted Clinical Likelihood model.’ Unfortunately, the two recommendations are not fully consistent with each other, and neither clearly define the abnormal clinical findings or specify whether the additional factors should increase or decrease the RF-CL—and, if so, by how much. The scoring system is evidence-based and quantitative up to a RF-CL estimate of 45% and then becomes semi-quantitative and subjective to the judgment of the treating physician, leaving the user uncertain on how to apply this in clinical practice.

For example, it is well-established that specific myocardial segments correspond to territories supplied by individual coronary arteries.^7^ From a pathophysiological perspective, it is therefore highly plausible that LV dysfunction can result from myocardial ischaemia caused by obstructive CAD. However, there is a significant distinction between LV dysfunction secondary to regional wall motion abnormalities corresponding to a coronary artery territory or global LV dysfunction.^8^ In the latter case, when combined with clinical findings such as atrial fibrillation, LV dysfunction could even lower the RF-CL.^9^

Moreover, listing of VA for RF-CL adjustment requires some degree of clarification as not all forms of VAs are related to structural heart disease like CAD.^10–12^ Patients without angina and benign VAs, such as young individuals with premature ventricular contractions and no CAD risk factors, should not be misclassified as suspected CCS.^11,12^

Listing *exercise ECG with abnormal findings* as an abnormal clinical finding qualifying for adjustment of the CL as a class I recommendation could also be difficult to interpret, as this non-invasive test is also part of the subsequent diagnostic pathway.^1,13^

### Step 3: sequential anatomical and functional testing

The ESC guidelines recommend ICA for patients with high-risk CAD on CCTA, severe ischaemia on functional imaging, refractory angina, or RF-CL above 85%. However, reaching this latter threshold is nearly impossible when starting from the RF-CL and subsequent adjustment based on abnormal clinical findings. For those with an adjusted RF-CL between >5% and ≤85%, a non-invasive imaging test is recommended. In selected cases, a sequential strategy using more than one non-invasive test might be necessary if findings remain ambiguous after initial tests. The post-test probability with the sequential testing strategy relies on a sophisticated calculation which requires pre-test probability estimates as well as positive and negative likelihood ratios for the non-invasive tests used, which depend on the respective test results. However, the exact methodology is not explained in the guideline document. Instead, it refers to the previous guideline version and other sources. From a practical perspective for the treating clinician, greater clarification on how to apply this using the guideline would be very helpful.

The lack of clear guidance has encouraged unofficial initiatives to close this gap. For example, a web application was recently disseminated through social media (e.g. https://ihtanboga2.shinyapps.io/CCS_app/) to estimate the RF-CL based on the regression formula published by Winther *et al*.^4^ The issue is that if one or more abnormal clinical findings is selected, the RF-CL is instantly adjusted to >85%, which is not supported by clinical or scientific evidence. Therefore, there is a need for professional societies to develop contemporary tools to further refine diagnostic test performance in individuals with suspected CCS.

Based on the RF-CL chart, we calculated post-test probabilities after a positive CCTA and an additional positive DSE in the second step (*Figure 4*). As can be seen, a CL >85% qualifying for ICA is not reached in any scenario after the first imaging test. Even after the second test, this threshold is only occasionally reached (primarily in older men with a symptom score of 2 to 3, presenting with atypical or typical angina). In some cases, even after two positive non-invasive tests which do not meet the criteria for direct ICA (i.e. high-risk CAD on CCTA or severe ischaemia on functional testing), the CL still does not exceed 85%. Therefore, it remains unclear to us how to proceed in such scenarios, if the guidelines are interpreted strictly algorithmically. In such situations, a pragmatic decision by the treating physician based on the individual clinical context is required.

The practical applicability of the 2024 ESC guidelines is further complicated by the fact that, while the document provides definitions for high-risk CAD on CCTA and/or severe ischaemia on functional testing that directly qualify for ICA, it does not clearly define what should be considered a positive, inconclusive, or negative test result for calculating the post-test CL, as illustrated in *Figure 4*. Although the positive and negative likelihood ratios are explicitly listed in the meta-analysis by Knuuti *et al*.,^13^ the corresponding definitions of a positive test must be retrieved from the original studies. In these, for example, a positive CCTA is often defined as a coronary stenosis of ≥50%, which clearly differs from the definition of high-risk CAD used in the guideline document.^14–17^ This definition would correspond to CAD-RADS 3.^18^ The prognostic significance of CCTA is undisputed, as the detection of even non-obstructive stenoses caused by atherosclerotic plaques triggers secondary prevention.^19^

Finally, this construct is based on studies applying a single-test strategy and has been extrapolated to a sequential test strategy.^13^ This would only be valid if the applied tests were independent of each other. However, this is unlikely to be the case in clinical practice, as the physician performing the second test will most likely know the results of the first test. For example, a DSE investigator might pay particular attention to the anterior wall segments if aware that the CCTA revealed a moderate stenosis in the LAD.

Routine application of the sequential testing strategy would dramatically increase the number of non-invasive tests to be performed. It is unclear whether there is sufficient capacity and expertise for these non-invasive tests. It should also be considered that the diagnostic studies—on which recommendations are based—were conducted at expert centres. If this approach was applied on a broader scale, it could result in many inconclusive test results, potentially leading to more rather than fewer ICAs. This would be neither medically nor economically reasonable. A routine sequential testing strategy carries the risk of overtesting, posing significant challenges to economy, ecology, and the patient, as nicely commented by Picano.^20^

Since physicians are subject to cognitive biases in diagnostic decision-making, and the tendency towards defensive medicine (e.g. due to fear of litigation) is well known, it would be highly desirable for the guidelines to prompt physicians to perform a well-founded risk–benefit assessment, which could be confidently communicated to justify this to their patients (especially when no test or no further test should be performed).^21^

In addition to the reclassification of the adjusted RF-CL based on all available clinical data, the ESC guidelines also recommend reclassification of the adjusted RF-CL according to coronary artery calcium scoring (CACS). Coronary artery calcium detected on a chest CT, performed for another indication, is usually assessed semi-quantitatively and cannot be converted into a CACS in routine clinical practice. For this scenario, the ESC guidelines define a corresponding abnormal clinical finding but do not specify to what extent the RF-CL should be increased based on this. Patients specifically undergoing coronary CT almost always undergo CACS first, followed by CCTA.

### Discussion of the case example

The patient in the case example in *Figure 1* of the Introduction has an ESC symptom score of 2 and, based on age, sex, and risk factor profile according to the RF-CL, an initial CL of 17%. The exercise ECG suggests exercise-induced ischaemia. This would likely have to be classified as ‘exercise ECG with abnormal findings’ according to Figure 5 of the guidelines for adjustment of the RF-CL. However, the guideline does not specify in which direction and to what extent the RF-CL should be adjusted. This is also inconsistent with the conclusion of the meta-analysis by Knuuti *et al*., which states that the ‘stress ECG appears to have limited diagnostic value at any level of pre-test probability’.^13^ Subsequently, two non-invasive tests are performed. Both show abnormal findings, but they neither meet the definition of high-risk CAD or severe ischaemia according to Table 14 of the guidelines. Nevertheless, one could decide to use these tests within the sequential testing strategy as outlined in Figures 7 and 8 of the guideline document. Using the positive likelihood ratios reported in the meta-analysis by Knuuti *et al*. to calculate the post-test probabilities, CLs of 48% and 81% are obtained. Ultimately, the patient therefore does not fulfil any criterion for invasive coronary angiography according to Table 11 of the guideline document (including a CL >85%). What would now be reasonable options? One approach would certainly be to state that every element of the mathematical model (from the initial RF-CL to the positive likelihood ratios of the diagnostic tests) is subject to statistical uncertainty, and that the threshold of CL >85% for proceeding to ICA was chosen arbitrarily. Thus, a pragmatic referral for ICA would be well justified. On the other hand, it would also be reasonable, after CCTA demonstrated obstructive CAD but no high-risk features, to end the diagnostic cascade and instead proceed with medical therapy alone (in particular secondary prevention of CAD with lipid-lowering therapy and aspirin). If angina pectoris were to recur, antianginal therapy could be initiated; should angina remain refractory despite this, proceeding to ICA would then be consistent with the guidelines.

### Summary and perspective

Although we strongly support the stepwise assessment of the CL as recommended by the 2024 ESC guidelines, some points would benefit from further clarification, as outlined above. While we acknowledge the positive aspects of the recommendations, particularly the emphasis on avoiding ICA by defining a sizeable population of individuals at very low risk of CAD, the rule-in capability remains a limitation of the current framework and relies on subsequent imaging tests. We therefore welcome the input from the PRO statement and hope this Great Debate can initiate a broader discussion on some fundamental aspects of the topic. On closer examination, it becomes apparent how little we truly know and how limited the data are for this core aspect in cardiology. We would like to advocate for the establishment of a large-scale and pragmatic registry performed by a collaborative European coronary diagnostics consortium. The goal would be to study the prevalence of obstructive CAD across Europe, gain deeper insights into the impact of findings potentially adjusting the CL, and validate the sequential testing procedure in contemporary cohorts.
