# Correction to: Great debate: the new risk factor–weighted clinical likelihood model is useful to estimate the initial pre-test probability of obstructive coronary artery disease in individuals with suspected chronic coronary syndromes

**DOI:** 10.1093/eurheartj/ehag457

**Published:** 2026-06-08

This is a correction to: Felicita Andreotti, Holger Thiele, Christiaan Vrints, Alexander Jobs, Simon Winther, Jens Gröninger, Davide Capodanno, Steffen Desch, Sotirios Nedios, Diana A. Gorog, Great debate: the new risk factor–weighted clinical likelihood model is useful to estimate the initial pre-test probability of obstructive coronary artery disease in individuals with suspected chronic coronary syndromes, *European Heart Journal,* Volume 47, Issue 22, 7 June 2026, Pages 2777-2792, https://doi.org/10.1093/eurheartj/ehag091

The following change has been made to the originally published paper.

The full author list and their respective affiliations list has been added at the top of this article.

