# Supplementary material for: Great debate: the new risk factor–weighted clinical likelihood model is useful to estimate the initial pre-test probability of obstructive coronary artery disease in individuals with suspected chronic coronary syndromes
Source: Eur Heart J. 2026 Mar 13;47(22):2777–92. doi: 10.1093/eurheartj/ehag091 (PMC13247188; doi:10.1093/eurheartj/ehag091)

## Supplemental Figure 1

Clinical likelihood of obstructive coronary artery disease (CAD) as chart tables in the temporal order of their publication. It is important to note that the chart tables differ in terms of the specified age strata. Additionally, for the risk factor-weighted clinical likelihood (RF-CL), the number of cardiovascular risk factors was introduced as an additional stratification variable. Risk factors include family history of CAD, defined as at least one first-degree relative with early signs of CAD (men under 55 and women under 65 years of age), smoking (current or past smoker), dyslipidaemia, hypertension, and diabetes. Furthermore, it should be noted that the qualitative description of symptoms as typical angina, atypical angina, and non-anginal chest pain in the RF-CL was replaced by a quantitative symptom score (3, 2, and 0 to 1 points, respectively). For the sake of consistency, we have retained the qualitative description in the figure. Additionally, it should be noted that, for reasons of simplicity and focus, we have omitted the probabilities for dyspnea as an angina equivalent, which were introduced in the chart tables from 2019 onward.


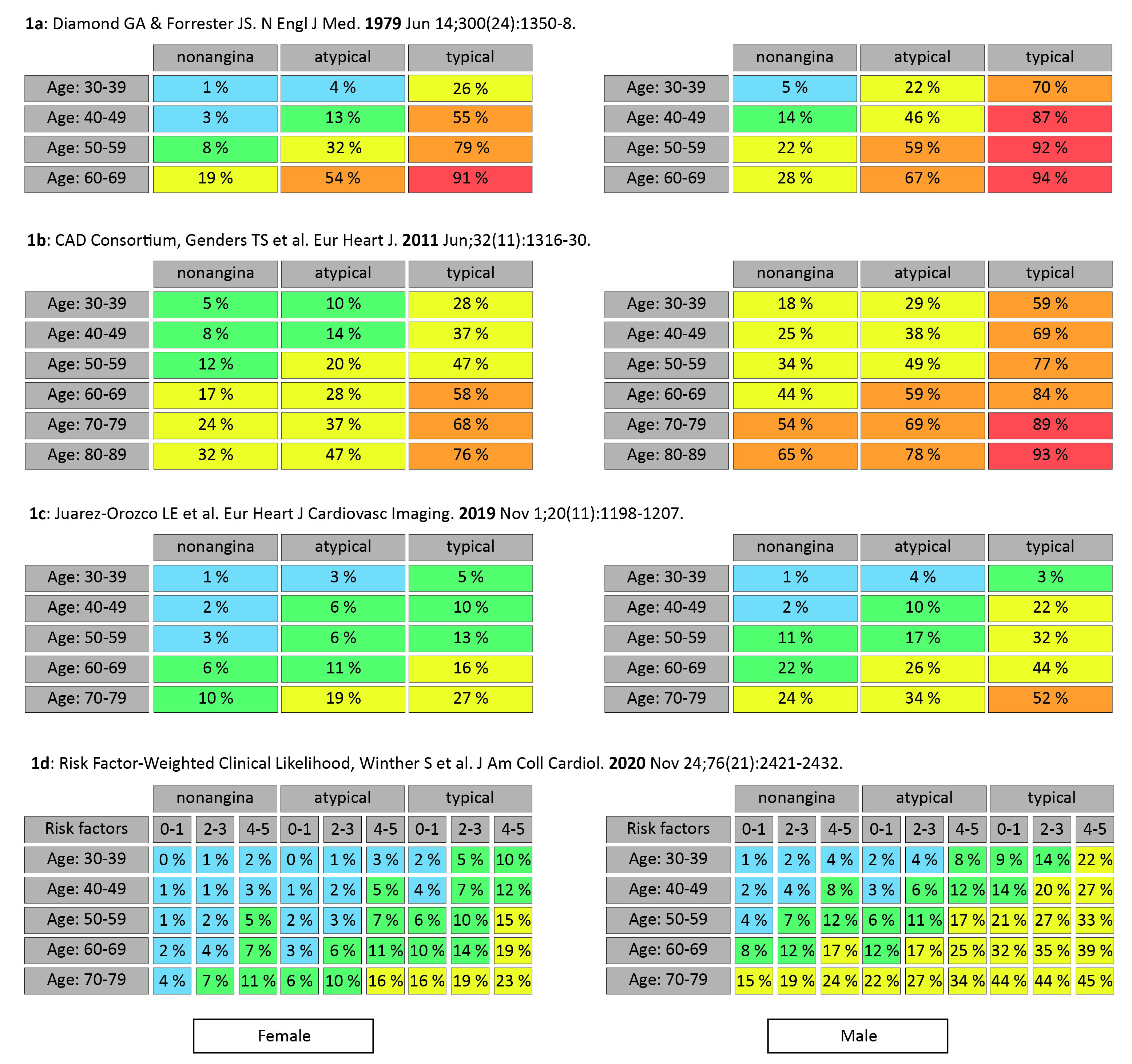

Supplement: ehag091_Supplementary_Data [file ehag091_supplementary_data.docx]
